# Antimicrobial Activity of D-Form Synthetic Peptides Against Metronidazole-Resistant and Susceptible *Trichomonas vaginalis*: A Comparative Transcriptomic Analysis

**DOI:** 10.3390/ijms27093747

**Published:** 2026-04-23

**Authors:** Özben Özden, Tuba Polat, Tanıl Kocagöz, Özgür Kurt

**Affiliations:** 1 Graduate School of Health Sciences, Department of Medical Biotechnology, Acibadem University, Istanbul 34752, Türkiye; tuubapolat24@gmail.com (T.P.); tanil.kocagoz@acibadem.edu.tr (T.K.); 2 Department of Medical Microbiology, Acibadem University School of Medicine, Istanbul 34752, Türkiye

**Keywords:** *Trichomonas vaginalis*, antimicrobial peptides, drug resistance, transcriptome, metronidazole

## Abstract

*Trichomonas vaginalis* is the causative agent of trichomoniasis, the most common non-viral sexually transmitted infection worldwide. In these cases, 5-Nitroimidazoles, particularly metronidazole (MTZ), remain the primary treatment option; however, resistance to MTZ has been increasingly reported. This study aimed to evaluate the in vitro activity of D-form synthetic antimicrobial peptides and investigate transcriptional differences associated with MTZ resistance and peptide treatment in *T. vaginalis*. D-form synthetic peptides (D-TN1, D-TN3, and D-TN6) developed in the R&D Laboratory of Acibadem University were tested against metronidazole-susceptible (*T. vaginalis* ATCC 30236) and metronidazole-resistant (*T. vaginalis* ATCC 50143) strains by minimum lethal concentration (MLC) assays. D-TN1 exhibited an MLC of 16 µg/mL in both strains, whereas D-TN3 and D-TN6 exhibited MLC values of 32 µg/mL and 16–32 µg/mL, respectively. Comparative transcriptomic analysis was conducted to investigate transcriptional differences. Differential gene expression analysis identified 3395 genes between the resistant and susceptible isolates and 3060 genes in the D-TN1-treated resistant isolate (FDR < 0.05, |log_2_FC| ≥ 1). D-TN1 treatment in the resistant isolate was associated with downregulation of ribosomal and metabolic pathways. If confirmed with further in vivo studies, this new antimicrobial peptide may become a new therapeutic alternative in the treatment of trichomoniasis in the future.

## 1. Introduction

Trichomoniasis is a common sexually transmitted infection caused by the flagellated protozoan, *Trichomonas vaginalis* [1,2]. According to the WHO, more than 156 million new cases are reported annually, while 5.3% of women worldwide are infected [3]. *T. vaginalis* colonizes mainly the urogenital tract in women, causing a spectrum of clinical manifestations ranging from asymptomatic colonization to vaginitis, cervicitis, and adverse outcomes during pregnancy [4]. Infected men are usually asymptomatic; however, various symptoms such as urethral discharge, dysuria, prostatitis, epididymitis, and infertility may be documented [2]. Yet, trichomoniasis in both sexes is mostly asymptomatic, and therefore its definitive diagnosis and treatment may be significantly limited in patients [5].

Metronidazole, a well-known 5-nitroimidazole derivative, has been the drug of choice for decades in trichomoniasis patients [6]. It shows cytotoxic effects in *T. vaginalis* after the reduction in hydrogenosomes over the low redox potential electron transport proteins, generating reactive nitro radicals that damage DNA and other macromolecules, leading to cell death. However, there is an emerging resistance and treatment failure against metronidazole in clinical isolates of *T. vaginalis* reported from different regions of the world [7]. Metronidazole resistance is associated with specific genetic and molecular characteristics of the parasite that causes impaired drug activation, which indicates the mutual contribution of the phenotypic and genotypic factors to reduced drug efficacy [8]. Therefore, both molecular and clinical studies need to be conducted together with systematic surveillance for the implementation of effective treatment strategies in trichomoniasis [9,10].

Antimicrobial peptides (AMPs) have emerged as potential therapeutic candidates due to their broad antimicrobial spectrum and diverse mechanisms. AMPs consist of several peptide families, including cathelicidins and defensins. Although conventional antibiotics are associated with metabolic pathways, antimicrobial peptides mainly interact with microbial membranes, resulting in membrane destabilization and loss of cellular integrity, while some peptides may also affect intracellular processes such as nucleic acid synthesis and protein production. Peptides synthesized in the D-amino acid form are less susceptible to protease degradation compared to L-form peptides, which improves their stability under biological conditions. D-TN peptides were previously shown to be completely resistant to proteases [11,12,13].

Studies have suggested that AMPs exhibit activity against parasites such as *Entamoeba histolytica*, *Giardia duodenalis*, *Trichomonas vaginalis*, *Trypanosoma cruzi*, *Leishmania tropica*, and *Plasmodium* spp. [14,15]. D-form peptides are considered more resistant to enzymatic degradation by host- and parasite-derived proteases. Consistent with this, D-hecate has been reported to exhibit anti-trichomonal activity, mainly through membrane disruption [16]. Synthetic antimicrobial peptides have demonstrated activity against *T. vaginalis*, including the metronidazole-resistant ATCC 50143 strain, with evidence indicating membrane damage [17].

This study aimed to evaluate the in vitro antiprotozoal activity of three D-form synthetic antimicrobial peptides (D-TN1, D-TN3, and D-TN6) by determining their minimum lethal concentrations, defined as the lowest concentration at which no viable trophozoites were observed after incubation, against metronidazole-susceptible (*T. vaginalis* ATCC 30236) and metronidazole-resistant (*T. vaginalis* ATCC 50143) strains.

These peptides have previously been investigated for their antibacterial and antifungal activities, and their safety profiles have been comprehensively evaluated, as reported by T. Kocagoz et al. (2025) [11]. In that study, in vitro analyses included cytotoxicity assessments in mammalian cell lines (mouse embryonic fibroblasts, 3T3, and human keratinocytes, HaCaT), hemolytic activity on human erythrocytes, and calculation of safety/selectivity indices. The results demonstrated low cytotoxicity toward mammalian cells and minimal hemolytic activity within the tested concentration range (0.5–32 μg/mL). Importantly, toxicity remained limited even at concentrations exceeding their antimicrobial effective levels, while IC_50_ was substantially higher than antibacterial and antifungal concentrations, indicating a safety profile and selective activity against microbial cells over host membranes. These peptides have well characterized antimicrobial activity and safety profiles; however, their potential against protozoan parasites has not yet been investigated [11]. In this study, their antiprotozoal activity was evaluated against *Trichomonas vaginalis.* In addition, comparative transcriptomic analysis was performed to identify gene expression differences between the resistant and susceptible isolates and to assess peptide-associated transcriptional responses in the resistant strain.

## 2. Results

**Figure 1:**
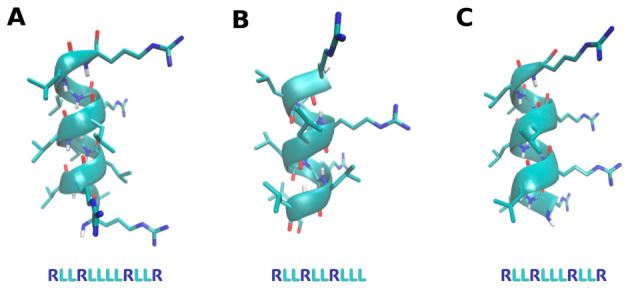
3D structure of the peptides. (**A**). D-TN1 (**B**). D-TN3 (**C**). DTN6. Atoms are colored as follows: H (white), O (red), N (blue), and C (cyan).

The antiprotozoal activities of the D-form antimicrobial peptides D-TN1 (RLLRLLLLRLLR), D-TN3 (RLLRLLRLLL), and D-TN6 (RLLRLLLRLLR), which exhibit α-helical three-dimensional structures as shown in Figure 1, were evaluated.

The D-form antimicrobial peptides were assessed in vitro against metronidazole-susceptible (MTZ-S) and metronidazole-resistant (MTZ-R) *T. vaginalis* isolates. The results are presented in Table 1. Metronidazole was used as a reference drug. The MLC of metronidazole was 1 µg/mL for the susceptible *T. vaginalis* strain, whereas it was >64 µg/mL for the resistant strain. D-TN1 exhibited the lowest MLC (16 µg/mL) against both isolates, indicating its potential as an antiprotozoal candidate. D-TN3 showed a higher MLC (32 µg/mL) against the resistant strain, and D-TN6 also showed a higher MLC (32 µg/mL), as shown in Table 1.

### 2.1. Differential Gene Expression Analysis (MTZ-R, S, and Peptide-Treated MTZ-R T. vaginalis)

Differential gene expression (DEGs) analysis identified 3395 genes between the metronidazole-resistant (ATCC 50143) and metronidazole-susceptible (ATCC 30236) strains, of which 2142 were upregulated in the susceptible strain and 1253 in the resistant strain (FDR < 0.05, |log_2_FC| ≥ 1). In the peptide-treated resistant isolate, 3060 differentially expressed genes were detected, including 1795 upregulated genes in the peptide-treated condition and 1265 genes upregulated in the untreated resistant isolate.

**Figure 2:**
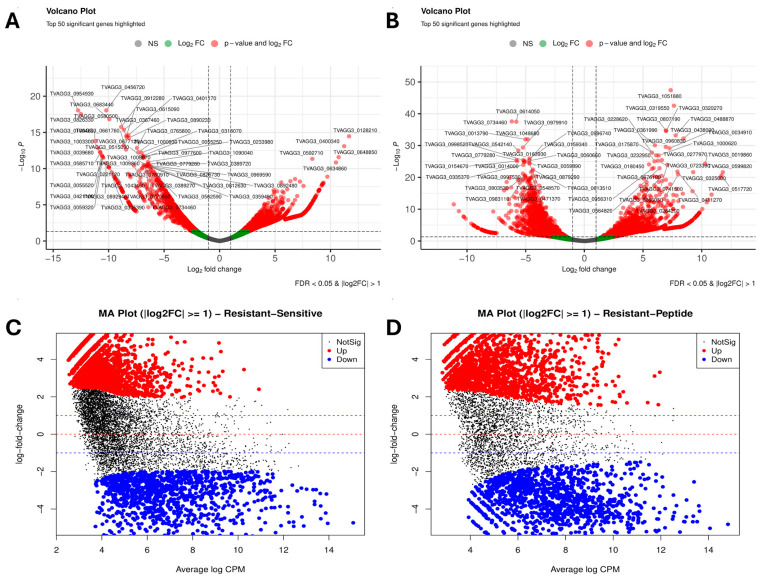
(**A**) Volcano plot of differentially expressed genes between MTZ-R and MTZ-S strains. (**B**) Volcano plot showing differentially expressed genes in the peptide-treated resistant strain. (**C**) MA plot comparing MTZ-R and MTZ-S strains. (**D**) MA plot of differential gene expression after peptide treatment.

DEGs identified several differentially expressed genes (FDR < 0.05, |log_2_FC| > 1). TVAGG3_0128210 (thioredoxin peroxidase; log_2_FC = 5.8, −log_10_p = 14.2), TVAGG3_0592710 (dehydrogenase 1-related family protein; log_2_FC = 6.9, −log_10_p = 11.57), and TVAGG3_0400340 (log_2_FC = 4.9, −log_10_p = 12.8) were upregulated in the resistant isolate as presented in Figure 2. However, TVAGG3_0954930 (FERM domain-containing protein; log_2_FC = −6.1, −log_10_p = 18.4), TVAGG3_0456720 (MCRA family protein; log_2_FC = −5.4, −log_10_p = 17.9), and TVAGG3_0826330 (cupin domain-containing protein; log_2_FC = −5.2, −log_10_p = 16.7) were significantly downregulated in the resistant isolate. The top dysregulated genes in peptide-treated MTZ-resistant *T. vaginalis* are shown in Table 2.

Volcano plot analysis of the peptide-treated metronidazole-resistant isolate revealed differential expression of several functionally relevant genes as detailed Figure 2. The most significantly upregulated transcripts were TVAGG3_1051880 and TVAGG3_0319550, both encoding GTPase, IMAP family-member-related proteins, as well as TVAGG3_0320270, encoding a thymidylate synthase family protein. Downregulation was observed for TVAGG3_0734460 (MIP02973P family protein), TVAGG3_0614050 (cytoskeletal anchoring protein), and TVAGG3_0998520 (pyruvate:ferredoxin oxidoreductase proprotein) as shown in Table 3. These genes exhibited high fold changes and statistical significance (FDR < 0.05; |log_2_FC| > 1), indicating transcriptional modulation after peptide treatment in the resistant strain.

**Figure 3:**
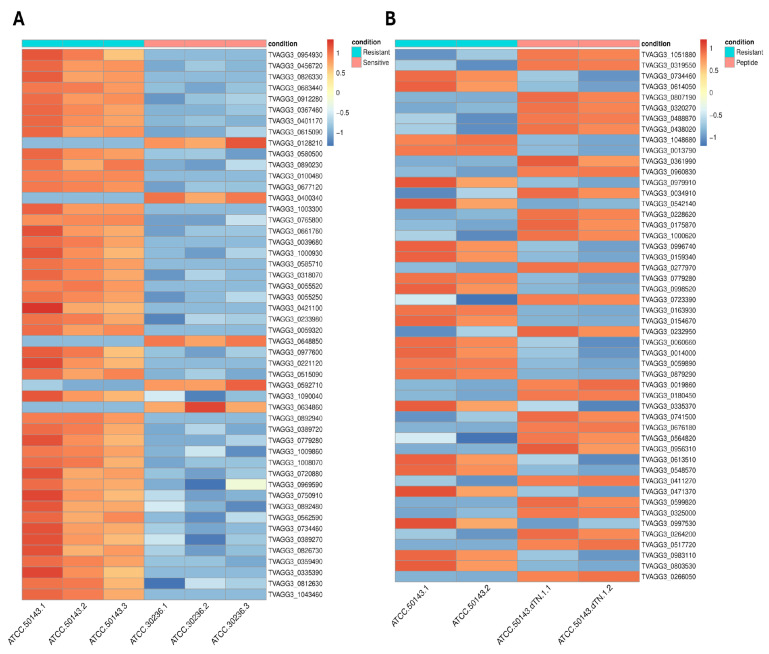
Heatmap of DEGs in *Trichomonas vaginalis*. (**A**) MTZ-R and MTZ-S strains (**B**) Peptide-treated resistant isolate. The color scale represents log2-transformed normalized expression values, with red indicating higher expression and blue indicating lower expression.

Heat map visualization of the top 50 differentially expressed genes revealed distinct transcriptional profiles between the experimental groups as shown in Figure 3. Heatmaps were generated using the heatmap package based on Z-score normalized CPM values to visualize gene expression patterns across sample groups. Each column represents an individual sample, and each row represents a differentially expressed gene. Gene expression values are shown as normalized and scaled expression levels, with red indicating relatively higher expression and blue indicating relatively lower expression across samples. Distinct expression patterns clearly separated metronidazole-resistant (MTZ-R) and metronidazole-susceptible (MTZ-S) strains, as well as peptide-treated and untreated resistant isolates. Replicates showed consistent transcriptional profiles.

### 2.2. Functional Enrichment Analysis (GO and KEGG) Resistant and Susceptible

**Figure 4:**
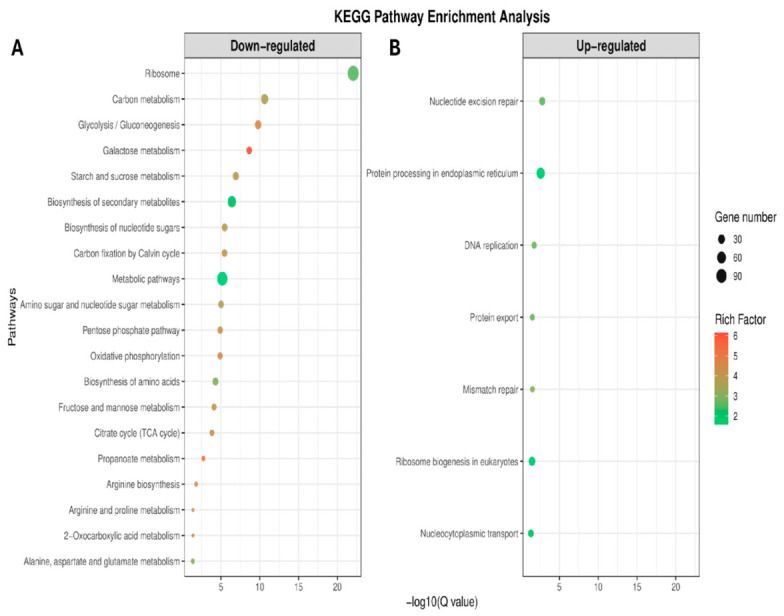
KEGG pathway enrichment analysis of (**A**) downregulated and (**B**) upregulated genes between MTZ-R and MTZ-S *T. vaginalis* strains. Dot size represents the number of genes associated with each pathway, and color indicates the rich factor.

Functional enrichment analysis of DEGs between resistant and susceptible strains identified significant enrichment of metabolic and cellular pathways. KEGG pathway enrichment analysis of differentially expressed genes identified a total of 27 significantly enriched pathways (FDR < 0.05), of which 7 were classified as upregulated and 20 as downregulated (Figure 4).

**Figure 5:**
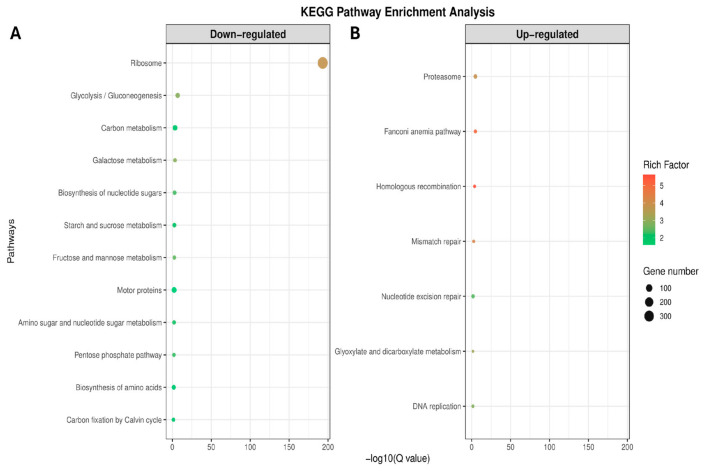
(**A**) Downregulated and (**B**) Upregulated KEGG pathways identified from the comparison with the peptide-treated metronidazole-resistant *T. vaginalis* isolate. Dot size represents the number of genes associated with each pathway, and color indicates the rich factor.

KEGG pathway enrichment analysis of peptide-treated DEGs identified 19 significantly enriched pathways (FDR < 0.05), including 7 upregulated and 12 downregulated pathways (Figure 5).

Downregulated genes were mainly enriched in pathways associated with central metabolism and energy production. The most significant enrichment was observed for ribosome (FDR = 9.1 × 10^−23^; gene count = 113), followed by carbon metabolism (FDR = 2.39 × 10^−11^) and glycolysis/gluconeogenesis (FDR = 1.65 × 10^−10^). Additional enrichment was detected in pathways related to carbohydrate metabolism, including galactose metabolism, starch and sucrose metabolism, and the pentose phosphate pathway. Other significantly enriched pathways included oxidative phosphorylation and the tricarboxylic acid (TCA) cycle.

Upregulated genes were mainly enriched in pathways associated with DNA and protein processing. These included nucleotide excision repair (FDR = 1.7 × 10^−3^), protein processing in the endoplasmic reticulum (FDR = 2.6 × 10^−3^), DNA replication (FDR = 1.82 × 10^−2^), and mismatch repair (FDR = 3.01 × 10^−2^). Additional enrichment was observed for ribosome biogenesis in eukaryotes and nucleocytoplasmic transport.

KEGG pathway enrichment analysis was subsequently performed to evaluate the transcriptional response of the resistant isolate after peptide treatment (Figure 5). Upregulated pathways were mainly associated with proteostasis and DNA repair mechanisms. The most significantly enriched pathway was the proteasome (FDR = 1.68 × 10^−5^; gene count = 17) and homologous recombination (FDR = 2.08 × 10^−4^). Additional enrichment was observed in mismatch repair (FDR = 2.60 × 10^−3^), nucleotide excision repair (FDR = 1.89 × 10^−2^), and DNA replication (FDR = 2.79 × 10^−2^).

A total of twelve pathways showed significant enrichment among downregulated genes. The most significant enrichment was detected for ribosome (FDR = 9.10 × 10^−23^; gene count = 113), followed by carbon metabolism (FDR = 2.39 × 10^−11^) and glycolysis/gluconeogenesis (FDR = 1.65 × 10^−10^). Additional enriched pathways included the pentose phosphate pathway (FDR = 1.28 × 10^−5^), oxidative phosphorylation (FDR = 1.30 × 10^−5^), and biosynthesis of amino acids (FDR = 5.07 × 10^−5^), indicating suppression of central metabolic and translational processes.

Gene ontology (GO) enrichment analysis was performed using a hypergeometric test with a significance threshold of FDR < 0.05. GO enrichment analysis of differentially expressed genes between the metronidazole-resistant and metronidazole-susceptible *T. vaginalis* strains as well as in the peptide-treated resistant group, revealed significant functional differences across biological process (BP), molecular function (MF), and cellular component (CC).

BP enrichment revealed significant involvement of the glycolytic process (GO:0006096), tricarboxylic acid cycle (GO:0006108), translation (GO:0006412), and protein folding (GO:0006457), indicating prominent alterations in central carbon metabolism and translational activity. BP was further assessed in the peptide-treated resistant group to determine peptide-treated alterations. It was mainly associated with translation (GO:0006412) and DNA repair (GO:0006281).

MF enrichment was observed for the structural constituent of ribosome (GO:0003735), RNA binding (GO:0003723), and ATP binding (GO:0005524), reflecting differential regulation of ribosomal components and energy-associated proteins between MTZ-resistant and susceptible strains. MF was further evaluated in the peptide-treated resistant group to identify functional changes associated with peptide treatment. It was observed for structural constituents of ribosome (GO:0003735), ATP binding (GO:0005524), and oxidoreductase activity (GO:0016491).

CC enrichment analysis showed ribosome (GO:0005840), cytoplasm (GO:0005737), mitochondrion (GO:0005739), and endoplasmic reticulum (GO:0005783) in the resistant versus susceptible comparison. CC enrichment was subsequently evaluated in the peptide-treated resistant isolate to determine structural changes associated with peptide treatment. CC enrichment analysis identified ribosome (GO:0005840), ribonucleoprotein complex (GO:0022625/GO:0022627), and mitochondrion (GO:0005739).

## 3. Discussion

Antimicrobial peptides have emerged as potential candidates due to their broad antimicrobial spectrum and mechanisms of action that differ from conventional drugs. Several studies have indicated that both natural and synthetic peptides are capable of reducing the viability and growth of *Trichomonas vaginalis*, including strains exhibiting decreased susceptibility to metronidazole. Peptide activity has been reported against both metronidazole-susceptible and resistant isolates, suggesting that these molecules can act independently of the metabolic pathways required for nitroimidazole activation [18].

C-terminally amidated tritrpticin (tritrpticin-NH_2_) is a tryptophan-rich antimicrobial peptide derived from porcine cathelicidin prophenin-2. The in vitro activity of tritrpticin-NH_2_ against *T. vaginalis* was evaluated for the first time. The results demonstrated a reduction in parasite viability and growth when the peptide was administered alone or in combination with metronidazole (tritrpticin-NH_2_ combined with low-concentration metronidazole, 5.8 μM) [19].

Studies investigated the propeptide form and hydroxylamine-cleaved prophenin-2. These studies showed disruption of *T. vaginalis* integrity and inhibition of parasite growth. Moreover, prophenin-2 exhibited partial resistance to proteolysis by *T. vaginalis* proteinases. Due to its relatively low hemolytic activity and short-term stability against parasite proteinases, prophenin-2 was proposed as a promising candidate for synergistic or alternative therapy against trichomoniasis [20].

Synthetic antimicrobial peptides have been developed. D-hecate is a 26-amino acid peptide derived from apitoxin. Ultrastructural analyses of parasites treated in vitro with 10 µM of D-hecate revealed extensive plasma membrane damage in both *T. foetus* and *T. vaginalis*, suggesting membrane disruption as a primary mechanism of action [16].

Antimicrobial peptides such as LL-37, KR-20, FK-13-NH_2_, and KR-12 have been evaluated against both metronidazole-susceptible (GT-13) and metronidazole-resistant (CDC-085) *T. vaginalis* strains. KR-20 showed MIC50 values of 4.8 µM for GT-13 and 7.8 µM for CDC-085, while LL-37, FK-13-NH_2_, and KR-12 also demonstrated inhibitory activity in both strains. In contrast, metronidazole exhibited an MIC50 of 5.8 nM in the susceptible strain and 2.8 µM in the resistant isolate [18].

The antiprotozoal activity of D-form synthetic peptides (D-TN1, D-TN3, and D-TN6) was evaluated against metronidazole-susceptible (ATCC 30236) and metronidazole-resistant (ATCC 50143) *T. vaginalis* strains in this study. Considering that D-TN1 exhibited in vitro MLC (16 µg/mL) in the resistant strain at concentrations substantially lower than MTZ, transcriptomic profiling was performed to explore the associated gene expression changes. However, MLC (16 µg/mL) is only two times lower than IC_50_ (32 µg/mL). Modification of D-TN1 should be considered to further increase the anti-trichomonal activity and lower the human cell cytotoxicity.

The most significantly upregulated genes in the metronidazole-resistant strain included TVAGG3_0592710, encoding a dehydrogenase-related protein, and TVAGG3_0120210, encoding thioredoxin peroxidase. Both genes are associated with redox regulation and oxidative stress response, indicating enhanced antioxidant capacity in the resistant isolate. In contrast, several genes were markedly downregulated, including TVAGG3_0954930 (FERM domain-containing protein), TVAGG3_0456720 (MCRA family protein), and TVAGG3_0826330 (cupin domain-containing protein), which are associated with cytoskeletal organization, membrane-associated processes, and metabolic enzyme activity. These changes suggest alterations in both structural and metabolic functions in the resistant strain.

Previous transcriptomic studies of resistant and susceptible *T. vaginalis* strains have reported altered expression of ribosomal proteins, carbohydrate metabolism-related genes, and redox-associated pathways, indicating that resistance is associated with stable transcriptional adaptation rather than acute drug-induced effects [21,22,23,24].

Consistent with these results, KEGG enrichment analysis in the present study showed that ribosome-related pathways, carbon metabolism, glycolysis/gluconeogenesis, the pentose phosphate pathway, and multiple amino acid metabolic pathways were downregulated in the resistant strain. Nucleotide excision repair, mismatch repair, and DNA replication pathways were enriched, suggesting increased genome maintenance activity. These results indicate reduced metabolic activity and increased DNA repair activity in the resistant strain.

In addition to these commonly reported features, pathways related to genome stability and protein processing were also affected, indicating that resistance is not limited to classical nitroimidazole-associated mechanisms.

In the peptide-treated resistant strain, suppression of ribosome-related processes and central carbon metabolism pathways was observed, together with enrichment of mismatch repair, nucleotide excision repair, and DNA replication pathways. This pattern is similar to the baseline resistant phenotype and suggests a common stress response characterized by reduced protein synthesis.

However, peptide treatment also led to distinct transcriptomic changes that were not present in the untreated resistant isolate. In particular, downregulation of the motor protein pathway was observed after peptide treatment. Motor proteins, including tva04814, are involved in flagellar movement, vesicle transport, and intracellular organization in *T. vaginalis.* The decreased expression of these genes suggests reduced motility and altered intracellular transport. This difference is important, as motility and intracellular transport can be related to parasite survival and host interaction.

These results suggest that metronidazole resistance is associated with a broad reduction in energy metabolism and biosynthetic capacity, while peptide treatment is associated with an additional layer of cellular stress, particularly affecting structural and motility-related systems which may contribute to the effectiveness of D-form antimicrobial peptides against metronidazole-resistant *T. vaginalis.*

The reliance on a single drug class (5-nitroimidazoles) for the treatment of trichomoniasis represents a therapeutic limitation in the context of emerging resistance [25]. Transcriptomic results further highlight the molecular complexity of the resistant phenotype and indicate the need for alternative treatment strategies aiming pathways beyond classical nitroimidazole activation mechanisms.

## 4. Materials and Methods

### 4.1. T. vaginalis Strain and Growth in Culture

*T. vaginalis* reference strains were obtained from the American Type Culture Collection (ATCC; Manassas, VA, USA). *T. vaginalis* strains used in this study were ATCC 50143, which is resistant to metronidazole, and ATCC 30236, which is susceptible to metronidazole. These reference strains were selected because they are widely used in antimicrobial resistance research, allowing standardized evaluation of peptide activity as well as direct comparison between metronidazole-susceptible and resistant isolates.

Trypticase yeast extract maltose (TYM) medium was used for the cultivation of *T. vaginalis* isolates. TYM medium was prepared by dissolving 20.0 g of trypticase, 10.0 g of yeast extract, 5.0 g of maltose, 1.0 g of L-cysteine HCl, 0.2 g of L-ascorbic acid, 0.2 g of KH_2_PO_4_, 0.2 g of K_2_HPO_4_, and 0.5 g of Bacto agar in 900 mL of distilled water. Firstly, 0.2 g of KH_2_PO_4_ and 0.2 g of K_2_HPO_4_ were added, and the other components were added sequentially under constant stirring with a magnetic stirrer. The pH was adjusted to 6.0 using 1 N HCl. The medium was sterilized at 121 °C for 15 min [26].

Before use, a sterile antibiotic solution (final concentrations: 10,000 units/mL penicillin and 10 mg/mL streptomycin) was added. When the medium was cooled to approximately 40 °C, 10% heat inactivated sterile horse serum (GIBCO^®^, Thermo-Fisher Ltd., Auckland, New Zealand) was aseptically added. The cultures were incubated at 37 °C and each strain was subcultured twice in fresh TYM medium to allow complete recovery and stabilization of growth before experimental use. At each passage, trophozoite viability and density were observed microscopically to confirm successful reactivation.

### 4.2. Preparation of Peptides

The antimicrobial peptides D-TN1 (RLLRLLLLRLLR), D-TN3 (RLLRLLRLLL), and D-TN6 (RLLRLLLRLLR) are structured as alpha-helices. D-amino acids were used to improve stability, as D-form peptides are more resistant to enzymatic degradation than L-form peptides. The 3D structures of the peptides were predicted by PEP-FOLD 4.0, and Model 1.1 was redrawn using VMD. The peptides were synthesized in the D-amino acid configuration using solid-phase peptide synthesis (SPPS) with a CEM Liberty Blue peptide synthesizer. SPPS was performed using standard Fmoc chemistry. The synthesized peptides were analyzed by reverse-phase high-performance liquid chromatography (RP-HPLC) using an analytical C18 column, and purity was confirmed to be greater than 95%. The peptides were dissolved in dH_2_O:ACN (7:3, *v*/*v*).

The antimicrobial peptides D-TN1 (RLLRLLLLRLLR), D-TN3 (RLLRLLRLLL), and D-TN6 (RLLRLLLRLLR) are short, cationic, and leucine-rich sequences with predicted amphipathic α-helical structures. Their main properties, including sequence, length, and net charge, are summarized in Table 4.

### 4.3. Susceptibility Testing for Metronidazole and D-Form Peptides

Their antibacterial and antifungal activities have already been identified, while no testing has yet been performed to assess their anti-protozoal activities. The anti-protozoal efficacies of these peptides were assessed in this study, against both metronidazole-susceptible and metronidazole-resistant isolates of reference *T. vaginalis* strains (ATCC 30236, ATCC 50143) in comparison with metronidazole.

The microdilution assay was performed in sterile, 96-well U-bottom cell culture plates. For this study, in order to prepare the inoculum containing protozoa at a standardized density, a hemocytometer was used for counting, and the suspension was adjusted to 1 × 10^4^ protozoa per mL. Then, 100 µL of *T. vaginalis* trophozoite was added to each well in trypticase yeast extract maltose medium. The stock peptides were diluted in TYM medium 128 to 0.25 µg/mL, and 100 µL was added to wells. Final concentrations of the peptide in the wells were adjusted to 64, 32, 16, 8, 4, 2, 1, 0.5, 0.25, and 0.125 µg/mL, respectively. For comparison, metronidazole was included as a reference drug. As a negative control, a sterile medium without peptide and trophozoites was added to one well to confirm medium sterility. As a positive growth control, trophozoites were added without peptide to verify the ability of the isolate to maintain viability. The experiment was performed in triplicate.

All plates were incubated at 37 °C and the trypan blue exclusion observed the growth of the parasites. After 48 h, each well was taken separately and mixed with trypan blue solution at a 1:1 ratio. The mixtures were then loaded onto a hemocytometer and examined under a microscope. Viable trophozoites were identified as unstained, whereas non-viable trophozoites took up the dye and appeared blue. The peptide concentration at which no viable trophozoites were observed was evaluated as the minimal lethal concentration.

### 4.4. RNA Extraction for RNA-Sequencing

Before RNA isolation, cell pellets were resuspended in TRI reagent and stored at −80 °C. Total RNA was extracted from the Qiagen RNeasy Mini Kit (Qiagen Gmbh, Hilden, Germany) according to the manufacturer’s instructions. The concentration and purity of the extracted RNA were assessed using a NanoDrop^TM^ spectrophotometer (Thermo-Fisher Ltd., Wilmington, DE, USA).

### 4.5. RNA Sequencing and Bioinformatic Analysis and Statistical Analysis

Reference strains of *Trichomonas vaginalis*, including the metronidazole-resistant ATCC 50143 strain, the MTZ-susceptible ATCC 30236 strain, and dTN1 synthetic antimicrobial peptide-treated experimental groups. Total RNA was extracted from parasite cultures using the Qiagen RNeasy Mini Kit (Qiagen Gmbh, Hilden, Germany), and RNA concentration and purity were assessed using a NanoDrop spectrophotometer (Thermo-Fisher Ltd., Wilmington, DE, USA). RNA sequencing libraries were prepared using the NEBNext^®^ Ultra™ II RNA Library Prep Kit for Illumina^®^ (New England Biolabs, Ipswich, MA, USA), including ribosomal RNA depletion, followed by cDNA synthesis, adapter ligation, and PCR amplification. Libraries were sequenced on the MGI DNBSEQ-T7 platform using paired-end 150 bp (PE150) reads. Raw reads were trimmed and quality-filtered using Cutadapt (https://doi.org/10.14806/ej.17.1.200), and sequencing quality was assessed with FastQC and MultiQC. Potential contamination was screened using Kraken2 (Johns Hopkins University Center for Computational Biology, Baltimore, MD, USA), and in silico rRNA depletion was performed using SortMeRNA (https://github.com/sortmerna/sortmerna, accessed on 1 April 2026). Cleaned reads were aligned to the *T. vaginalis* reference genome (TrichDB v51) using HISAT2 (https://daehwankimlab.github.io/hisat2/, accessed on 1 April 2026), followed by post-alignment processing with Samtools. Gene level read counts were generated using featureCounts (https://subread.sourceforge.net/featureCounts.html, accessed on 1 April 2026). Differential gene expression analysis was conducted using the edgeR package with TMM normalization, and genes with a false discovery rate (FDR) < 0.05 and |log_2_ fold change| ≥ 1.0 were considered statistically significant. Principal component analysis (PCA), heatmaps, and volcano plots were generated in R (https://cran.r-project.org/, accessed on 1 April 2026) using ggplot2, pheatmap, and EnhancedVolcano. Functional enrichment analyses for gene ontology terms and KEGG pathways were performed using clusterProfiler (https://bioconductor.org/packages/release/bioc/html/clusterProfiler.html, accessed on 1 April 2026) and KEGGREST (https://bioconductor.posit.co/packages/3.19/bioc/html/KEGGREST.html, accessed on 1 April 2026).

## 5. Conclusions

Transcriptomic analysis revealed differences in gene expression between metronidazole-resistant and metronidazole-susceptible *T. vaginalis* strains. The resistant strain showed reduced expression of metabolic and translational pathways, including ribosome, glycolysis, and carbon metabolism, together with increased expression of DNA repair and stress-related pathways, indicating a distinct transcriptional profile associated with resistance.

Peptide treatment of the resistant strain resulted in both shared and distinct transcriptomic changes, identified by activation of proteasome-related pathways that were specifically enriched. In contrast, suppression of motor-protein-related processes suggested impaired parasite motility and altered intracellular organization.

This study has several limitations that should be considered when interpreting the results. First, the analyses were performed using only two reference strains of *T. vaginalis,* representing MTZ-R and MTZ-S phenotypes. Future studies including a larger number of clinical samples are needed to validate these results. Furthermore, the pharmacokinetic properties and in vivo efficacy of these peptides need to be evaluated. Integration of proteomic analyses would provide a more comprehensive understanding of the biological processes involved.

Despite these limitations, studies investigating the antiprotozoal activity of antimicrobial peptides, particularly at the transcriptomic analysis, remain limited, and this study represents a valuable contribution in this field.

## Figures and Tables

**Table 1:** Minimum lethal concentration of the peptides and metronidazole against metronidazole-susceptible and metronidazole-resistant *T. vaginalis* strains.

Peptides	ATCC 50143 (MTZ Resistant) MLC	ATCC 30236 (MTZ Susceptible) MLC
**DTN1**	16 µg/mL	16 µg/mL
**DTN3**	32 µg/mL	16 µg/mL
**DTN6**	32 µg/mL	32 µg/mL
**Antibiotic (reference)**		
**Metronidazole**	>64 µg/mL	1 µg/mL

**Table 2:** Top dysregulated genes in MTZ-R and MTZ-S *T. vaginalis.* Most significantly differentially expressed genes (FDR < 0.05 & |log_2_FC| > 1).

Gene ID	log_2_FC	−log_10_(p)
TVAGG3_0128210	5.8	14.2
TVAGG3_0592710	6.9	11.5
TVAGG3_0400340	4.9	12.8
TVAGG3_0954930	−6.1	18.4
TVAGG3_0456720	−5.4	17.9
TVAGG3_0826330	−5.2	16.7

**Table 3:** Top dysregulated genes in peptide-treated MTZ-resistant *T. vaginalis.* Most significantly differentially expressed genes (FDR < 0.05 & |log_2_FC| > 1).

Gene ID	log_2_FC	−log_10_(p)
TVAGG3_1051880	8.7	47.5
TVAGG3_0319550	7.9	44.2
TVAGG3_0320270	6.8	41.0
TVAGG3_0734460	−8.3	37.6
TVAGG3_0614050	−7.5	39.8
TVAGG3_0998520	−6.4	32.7

**Table 4:** Physicochemical properties of peptides.

Peptide	Sequence	Length	Net Charge	Hydrophobic Residues (%)	Isoelectric Point (pI)	Molecular Mass (g/mol)
D-TN1	RLLRLLLLRLLR	12	+6	33	12.96	1547.05
D-TN3	RLLRLLRLLL	10	+4	30	12.79	1277.71
D-TN6	RLLRLLLRLLR	11	+5	36	12.96	1433.89

## Data Availability

The original contributions presented in this study are included in the article/Supplementary Material. Further inquiries can be directed to the corresponding author.

## Supplementary Materials

The following supporting information can be downloaded at: https://www.mdpi.com/article/10.3390/ijms27093747/s1.

## Abbreviations

The following abbreviations are used in this manuscript:

AMPs	Antimicrobial peptides
DEGs	Differential gene expression
FDR	False discovery rate
GO	Gene Ontology
KEGG	Kyoto Encyclopedia of Genes and Genomes
MLC	Minimum lethal concentration
MTZ	Metronidazole
MTZ-S	Metronidazole susceptible
MTZ-R	Metronidazole resistance
TYM	Tripticase-Yeast-Exctract-Maltose
WHO	World Health Organization
