# Supplementary material for: Antimicrobial Activity of D-Form Synthetic Peptides Against Metronidazole-Resistant and Susceptible Trichomonas vaginalis: A Comparative Transcriptomic Analysis
Source: Int J Mol Sci. 2026 Apr 23;27(9):3747. doi: 10.3390/ijms27093747 (PMC13164389; doi:10.3390/ijms27093747)
Supplement: Supplementary file 1 [file ijms-27-03747-s001.zip › Figure S2_MTZ_Resistant-Sensitive_volcano_plot_no_overlap.pdf]

Volcano Plot

Top 50 significant genes highlighted

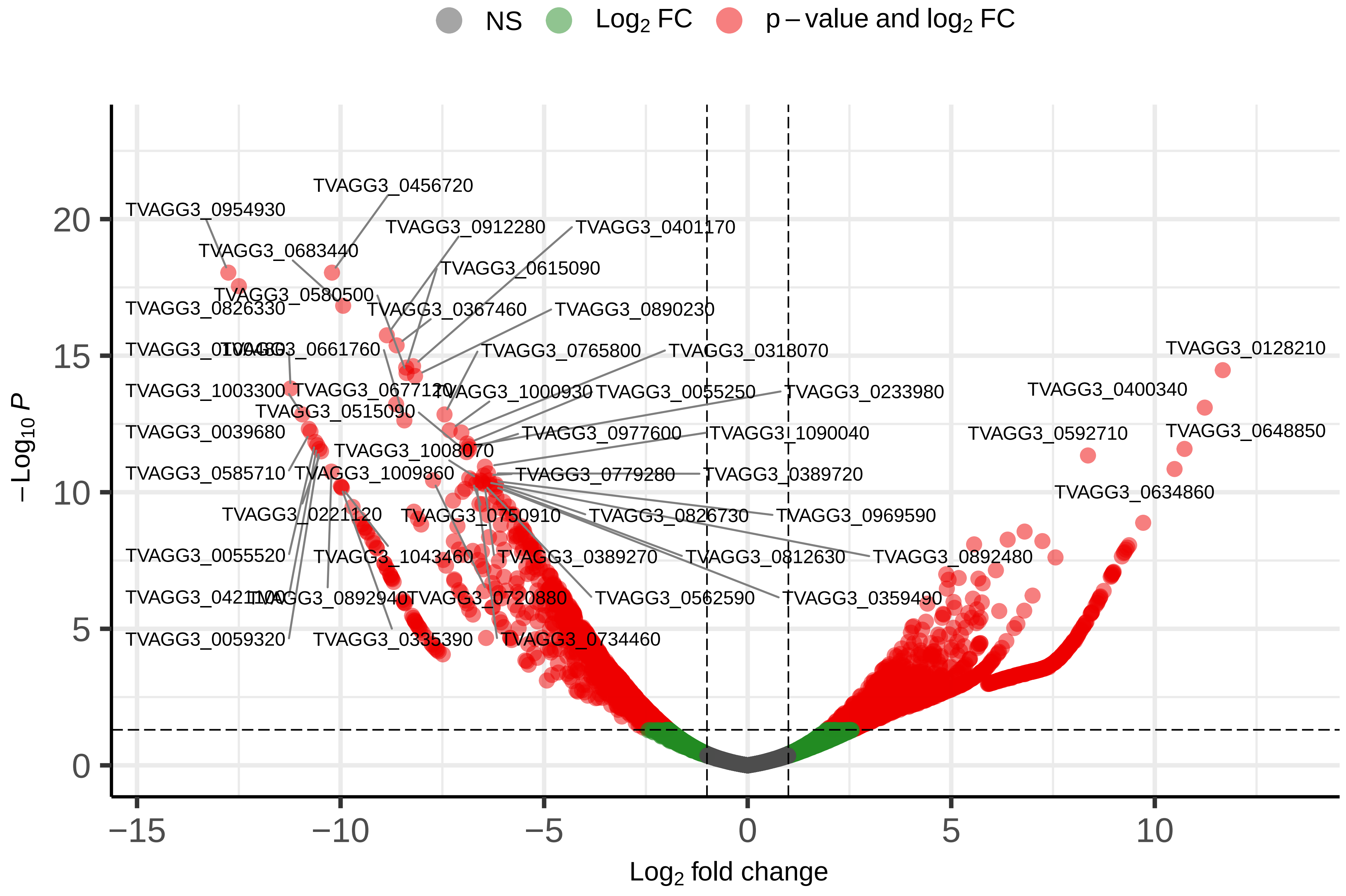

FDR < 0.05 & |log2FC| > 1
