# Supplementary material for: Antimicrobial Activity of D-Form Synthetic Peptides Against Metronidazole-Resistant and Susceptible Trichomonas vaginalis: A Comparative Transcriptomic Analysis
Source: Int J Mol Sci. 2026 Apr 23;27(9):3747. doi: 10.3390/ijms27093747 (PMC13164389; doi:10.3390/ijms27093747)
Supplement: Supplementary file 1 [file ijms-27-03747-s001.zip › Figure S3_GO_Dotplot_Resistant-Peptide.pdf]

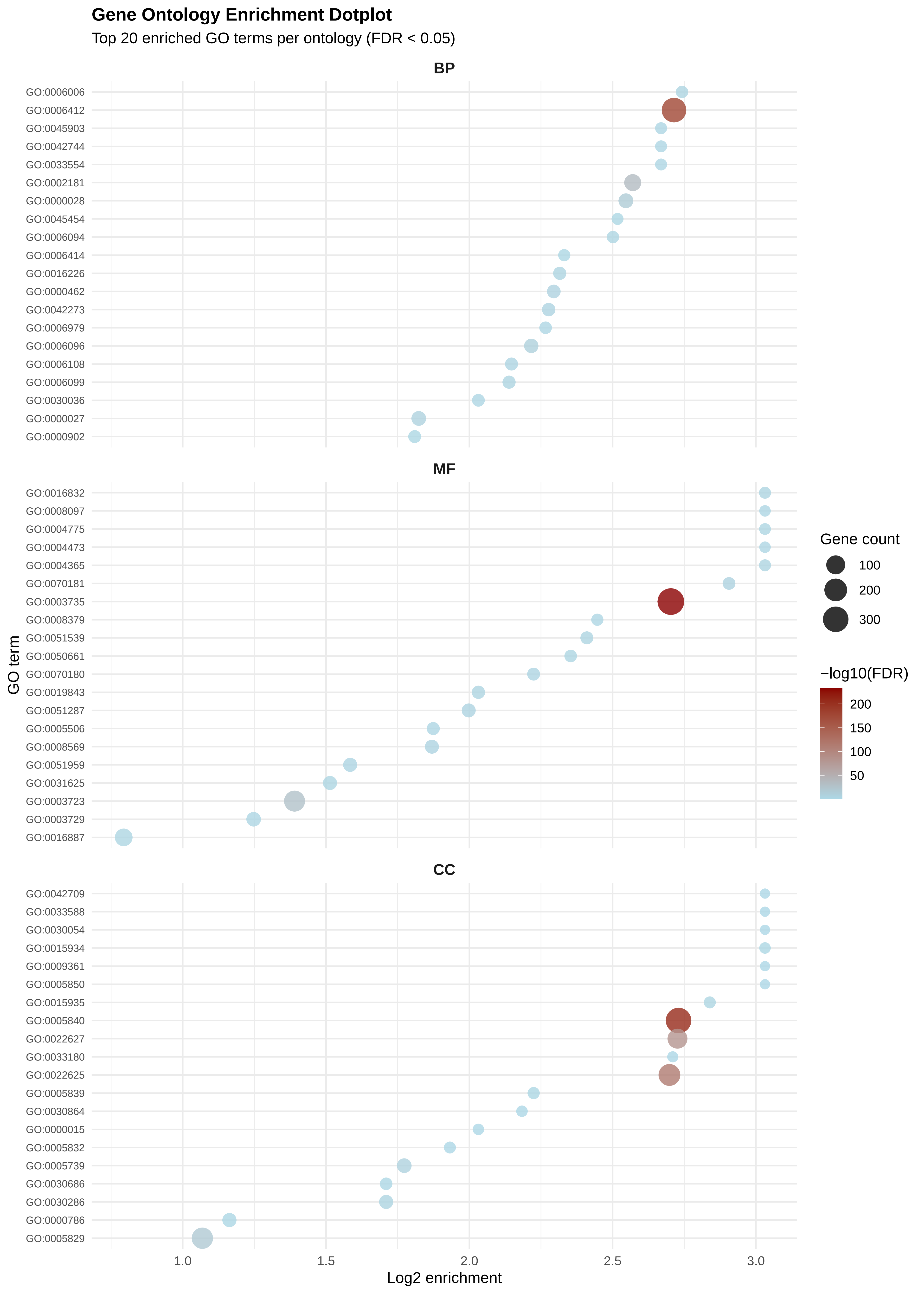

Gene Ontology Enrichment Dotplot
Top 20 enriched GO terms per ontology (FDR < 0.05)
BP
GO:0006006
GO:0006412
GO:0045903
GO:0042744
GO:0033554
GO:0002181
GO:0000028
GO:0045454
GO:0006094
GO:0006414
GO:0016226
GO:0000462
GO:0042273
GO:0006979
GO:0006096
GO:0006108
GO:0006099
GO:0030036
GO:0000027
GO:0000902
MF
GO:0016832
GO:0008097
GO:0004775
GO:0004473
GO:0004365
GO:0070181
GO:0003735
GO:0008379
GO:0051539
GO:0050661
GO:0070180
GO:0019843
GO:0051287
GO:0005506
GO:0008569
GO:0051959
GO:0031625
GO:0003723
GO:0003729
GO:0016887
CC
GO:0042709
GO:0033588
GO:0030054
GO:0015934
GO:0009361
GO:0005850
GO:0015935
GO:0005840
GO:0022627
GO:0033180
GO:0022625
GO:0005839
GO:0030864
GO:0000015
GO:0005832
GO:0005739
GO:0030686
GO:0030286
GO:0000786
GO:0005829
GO term
1.0
1.5
2.0
2.5
3.0
Log2 enrichment
Gene count
100
200
300
−log10(FDR)
200
150
100
50
