# Supplementary figures and images for: Antimicrobial Activity of D-Form Synthetic Peptides Against Metronidazole-Resistant and Susceptible Trichomonas vaginalis: A Comparative Transcriptomic Analysis
Source: Int J Mol Sci. 2026 Apr 23;27(9):3747. doi: 10.3390/ijms27093747 (PMC13164389; doi:10.3390/ijms27093747)

### Top 50 significant genes highlighted

### Top 50 significant genes highlighted

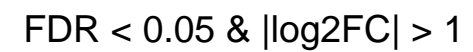

Supplement: Supplementary file 1 [file ijms-27-03747-s001.zip › Figure S1 Resistant-Peptide_volcano_plot_no_overlap.pdf]

Gene Ontology Enrichment Dotplot

Top 20 enriched GO terms per ontology (FDR < 0.05)

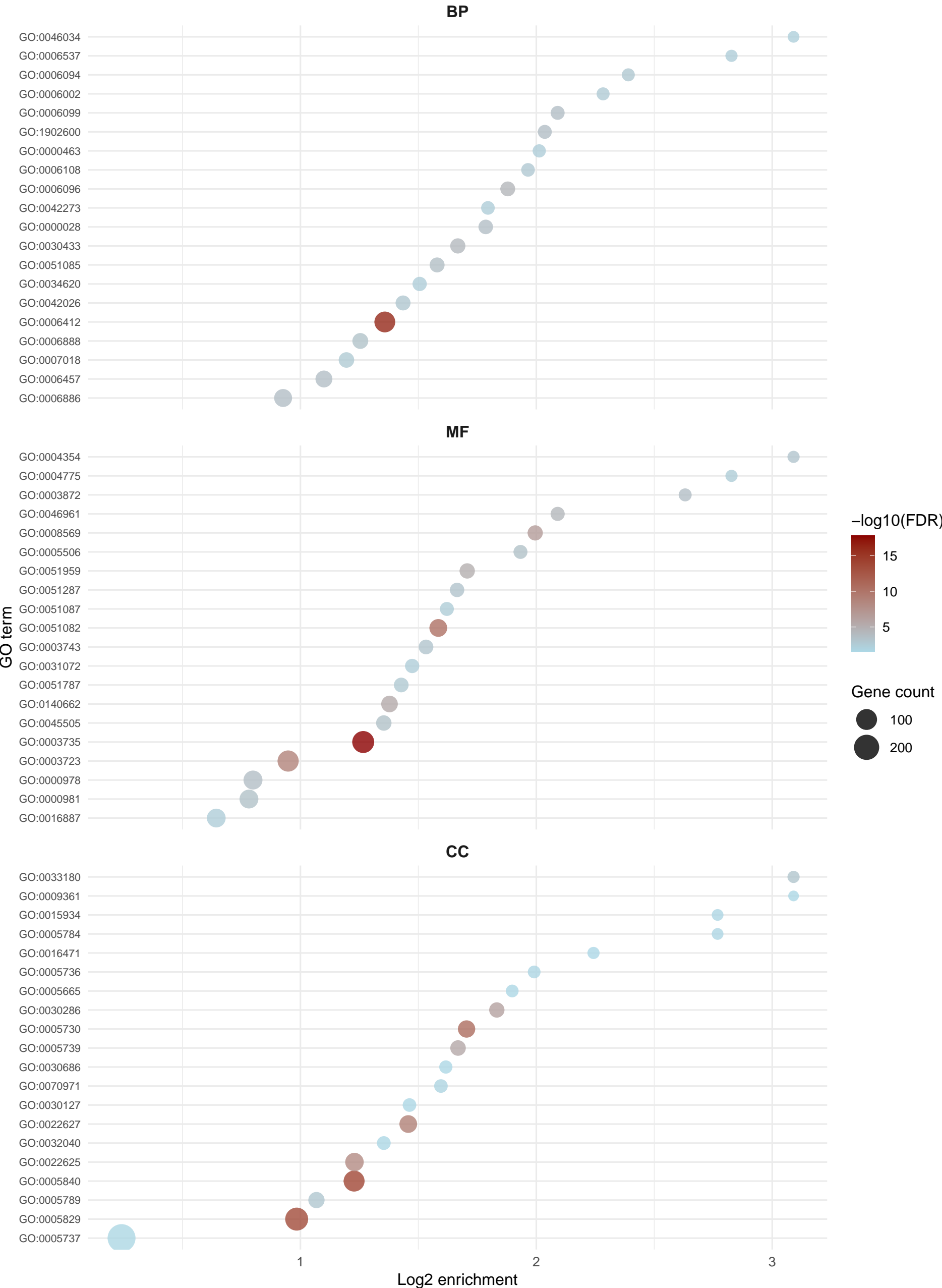

Supplement: Supplementary file 1 [file ijms-27-03747-s001.zip › Figure S4_GO_Dotplot_Resistant-Sensitive.pdf]

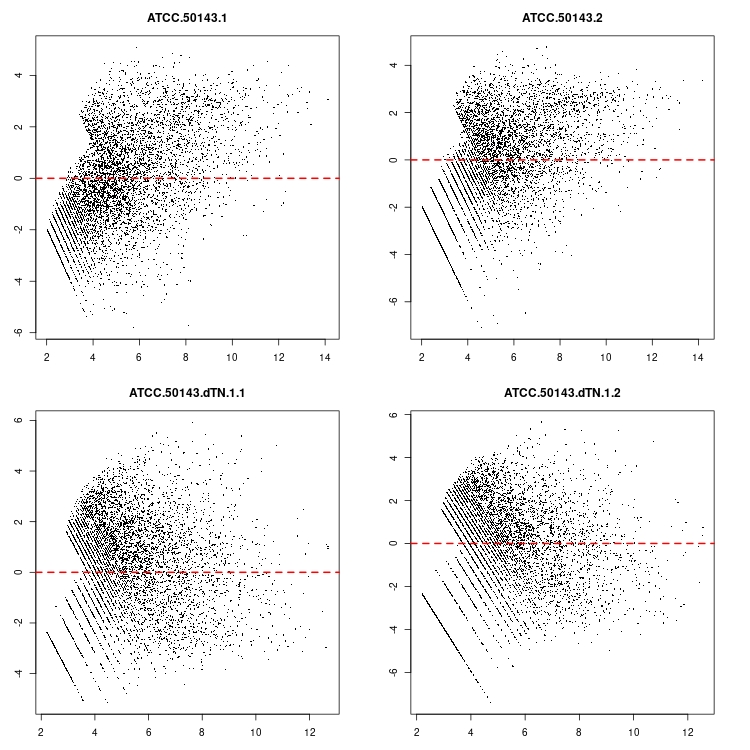

Supplement: Supplementary file 1 [file ijms-27-03747-s001.zip › Figure S5_MDPlots_Resistant-Peptide.png]

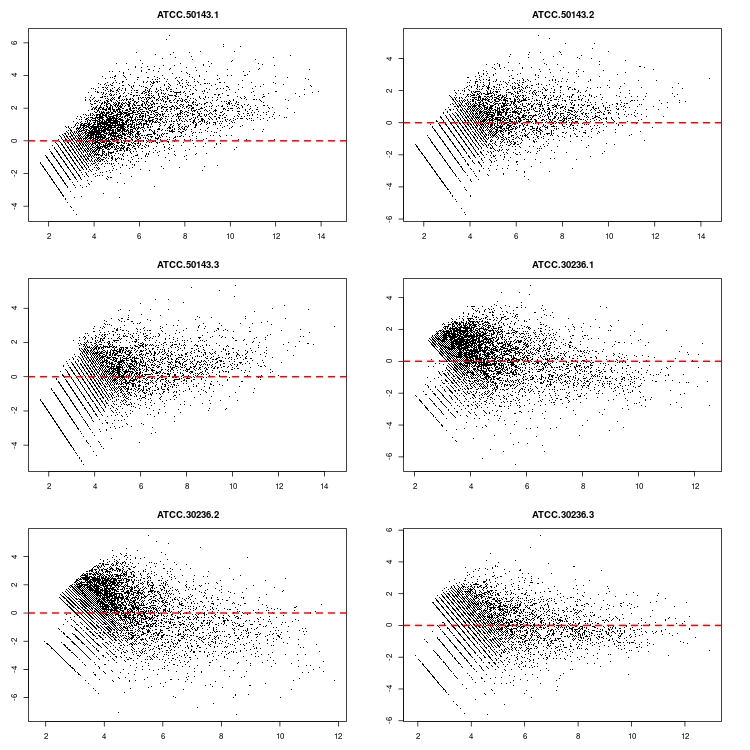

Supplement: Supplementary file 1 [file ijms-27-03747-s001.zip › Figure S6_MDPlots_MTZ_Resistant-Sensitive.png]
